# Investigating the impact of COVID-19 lockdown on adults with a recent history of recurrent major depressive disorder: a multi-Centre study using remote measurement technology

**DOI:** 10.1186/s12888-021-03434-5

**Published:** 2021-09-06

**Authors:** Daniel Leightley, Grace Lavelle, Katie M. White, Shaoxiong Sun, Faith Matcham, Alina Ivan, Carolin Oetzmann, Brenda W. J. H. Penninx, Femke Lamers, Sara Siddi, Josep Mario Haro, Inez Myin-Germeys, Stuart Bruce, Raluca Nica, Alice Wickersham, Peter Annas, David C. Mohr, Sara Simblett, Til Wykes, Nicholas Cummins, Amos Akinola Folarin, Pauline Conde, Yatharth Ranjan, Richard J. B. Dobson, Viabhav A. Narayan, Mathew Hotopf, On behalf of the RADAR-CNS Consortium

**Affiliations:** 1 Department of Psychological Medicine, Institute of Psychiatry, Psychology and Neuroscience, King’s College London, London, UK; 2 Department of Biostatistics and Health Informatics, Institute of Psychiatry, Psychology and Neuroscience, King’s College London, London, UK; 3 Department of Psychiatry, Amsterdam UMC, Vrije Universiteit, Amsterdam, The Netherlands; 4 Parc Sanitari Sant Joan de Déu, Fundació Sant Joan de Déu, Barcelona, Spain; 5 Universitat de Barcelona, Barcelona, Spain; 6 Centro de Investigación Biomédica en Red de Salud Mental, CIBERSAM, Madrid, Spain; 7 Center for Contextual Psychiatry, Department of Neurosciences, KU Leuven, Leuven, Belgium; 8 RADAR-CNS Patient Advisory Board, King’s College London, London, UK; 9 Romanian League for Mental Health, London, UK; 10 H. Lundbeck A/S, Copenhagen, Denmark; 11 Center for Behavioral Intervention Technologies, Northwestern University, Chicago, USA; 12 King’s College London, Institute of Psychiatry, Psychology & Neuroscience, London, UK; 13 Chair of Embedded Intelligence for Health Care & Wellbeing, University of Augsburg, Augsburg, Germany; 14 South London and Maudsley NHS Foundation Trust, London, UK; 15 Institute of Health Informatics, University College London, London, UK; 16 Maudsley Biomedical Research Centre, National Institute for Health Research, South London and Maudsley NHS Foundation Trust, London, UK; 17 Janssen Research and Development, LLC, Titusville, NJ USA

**Keywords:** Remote measurement technology, Major depressive disorder, Mobile health

## Abstract

**Background:**

The outbreak of severe acute respiratory syndrome coronavirus 2 (SARS-CoV-2), which causes a clinical illness Covid-19, has had a major impact on mental health globally. Those diagnosed with major depressive disorder (MDD) may be negatively impacted by the global pandemic due to social isolation, feelings of loneliness or lack of access to care. This study seeks to assess the impact of the 1st lockdown – pre-, during and post – in adults with a recent history of MDD across multiple centres.

**Methods:**

This study is a secondary analysis of an on-going cohort study, RADAR-MDD project, a multi-centre study examining the use of remote measurement technology (RMT) in monitoring MDD. Self-reported questionnaire and passive data streams were analysed from participants who had joined the project prior to 1st December 2019 and had completed Patient Health and Self-esteem Questionnaires during the pandemic (*n* = 252). We used mixed models for repeated measures to estimate trajectories of depressive symptoms, self-esteem, and sleep duration.

**Results:**

In our sample of 252 participants, 48% (*n* = 121) had clinically relevant depressive symptoms shortly before the pandemic. For the sample as a whole, we found no evidence that depressive symptoms or self-esteem changed between pre-, during- and post-lockdown. However, we found evidence that mean sleep duration (in minutes) decreased significantly between during- and post- lockdown (− 12.16; 95% CI − 18.39 to − 5.92; *p* <  0.001). We also found that those experiencing clinically relevant depressive symptoms shortly before the pandemic showed a decrease in depressive symptoms, self-esteem and sleep duration between pre- and during- lockdown (interaction *p* = 0.047, *p* = 0.045 and *p* <  0.001, respectively) as compared to those who were not.

**Conclusions:**

We identified changes in depressive symptoms and sleep duration over the course of lockdown, some of which varied according to whether participants were experiencing clinically relevant depressive symptoms shortly prior to the pandemic. However, the results of this study suggest that those with MDD do not experience a significant worsening in symptoms during the first months of the Covid − 19 pandemic.

**Supplementary Information:**

The online version contains supplementary material available at 10.1186/s12888-021-03434-5.

## Background

On the 31st December 2019, the World Health Organisation (WHO) documented reports of a cluster of cases of pneumonia of unknown origin in Wuhan, China [1]. The cause was later identified as a novel severe acute respiratory syndrome coronavirus 2 (SARS-CoV-2), which caused a clinical illness, Covid-19 [2]. Within weeks of the initial outbreak, the total number of cases and deaths had exceeded those of severe acute respiratory syndrome outbreak in 2003 [3]. In March 2020, a global pandemic was declared by the WHO due to the exponential increase in diagnosed cases and deaths, with countries across Europe implementing national lockdowns to reduce the risk of spread and infection [4].

The ongoing Covid-19 pandemic is predicted to have severe negative global mental health consequences [5, 6], with a review of stressors indicating that quarantine duration, infection fears, frustration, boredom, inadequate information, financial loss, loss of sleep and stigma being the main drivers [7]. The pandemic has disrupted or halted critical mental health services in 93% of countries worldwide, while the demand for mental health care is increasing, according to a recent WHO survey [8]. There have been urgent calls to examine the mental health consequences of Covid-19 at an international level, using high-quality data and robust analysis techniques [9–11].

Gaining a clear indication of population impacts of the pandemic on mental health has been challenging [6]. Pre-existing population studies, which have explicit sampling frames and longitudinal data pre-dating the pandemic, have demonstrated an increase in symptoms of distress within the general population [6, 12]. And in the early stages of the pandemic this was dominated by symptoms of anxiety [12], with symptoms of distress most frequent in young adults [6]. In addition, one population study found sleep to be negatively impacted by the pandemic, with female participants reporting more sleep loss than male participants [13].

A less studied issue has been the impact of public health measures, such as lockdown, on individuals with pre-existing mental disorders who may have less access to care and support services [8]. Adults diagnosed with major depressive disorder (MDD) and experiencing a current episode of depression are particularly susceptible to the challenges raised by lockdown, such as disrupted sleep [14], reduced sociability [15] and changes in mood/self-esteem [16]. Therefore, it is important to understand the trajectories of change in those experiencing a current episode of depression and how these outcomes are impacted by the pandemic.

Across Europe, smartphone ownership and use is high (estimated 76% of adults across Europe [17]), which provides a ready means for accurate and ongoing data collection using remote measurement technology (RMT) [18–20]. RMT data collection methods are inexpensive, can gather data in real-time, and crucially considering infection risk, do not require face-to-face contact between the research team and participants. RMT may provide a solution to the need for surveillance at the population level passively, without the need for intrusive study protocols, or continual engagement. This may lead to richer, more objective and holistic characterisation of behaviours and physiology as a result of the Covid-19 pandemic.

Remote Assessment of Disease and Relapse in individuals with Major Depressive Disorder (RADAR-MDD) is an ongoing study forming a component of the RADAR-Central Nervous System consortium [21]. Participants with MDD from the UK, Spain and The Netherlands were invited to provide longitudinal data via ubiquitous, commercially-available RMT (i.e. phones and activity trackers) [18]. The high-frequency information collected passively includes detail on participants’ sleep quality/patterns, physical activity, stress, mood, self-esteem, sociability, speech patterns, and cognitive function [18]. In addition to passive data collection, self-reported ecological momentary assessment data was also collected. This included assessments focusing on depression, speech, self-esteem, and cognitive function. The study provides an opportunity to explore the impact of the pandemic on individuals with a MDD diagnosis and their changes in depressive symptoms across Europe. A strength of RADAR-CNS is the ability to directly compare results gathered during- and post- lockdown with previously collected pre-lockdown baseline data.

The potential impact of the pandemic on individuals with mental disorders has been recognised as one of a triad of key current global mental health challenges [22]. Relatively high rates of depression have been reported by a number of countries [23], this adding to the existing global burden of depression [24]. However, tackling this in its entirety demands a greater understanding of the true impact of Covid-19 for those living with pre-existing mental disorders. We therefore aimed to investigate the impact of the 1st global lockdown on adults with a recent history of MDD, through the following objectives: 1) To investigate changes in depressive symptoms, self-esteem and sleep duration pre-, during- and post-lockdown in the period from 1st December 2019 to 1st September 2020; and 2) To investigate whether these changes over time varied according to whether participants were experiencing a depressive episode shortly before the pandemic.

## Method

### Data source and participants

This study uses data collected between 1st December 2019 and 1st September 2020 (9 months of available data) from the RADAR-MDD project, a multi-centre cohort, examining the use of RMT in monitoring MDD [18]. Participants were required to meet the following eligibility criteria: 1) DSM-5 diagnostic criteria for diagnosis of non-psychotic MDD in the last 2 years, 2) recurrent MDD (lifetime history of at least 2 episodes of depression, 3) willingness and ability to complete self-reported assessment via smartphone, 4) provide informed consent, 5) own an Android smartphone, or willing to use an Android smartphone provided by the research team, 6) aged 18 years or over, and 7) fluent in English, Spanish, Catalan or Dutch. The study protocol for RADAR-MDD has been previously reported [18].

The data collected via RADAR-MDD project uses RADAR-base, which is an open source platform designed to leverage data from wearables and mobile technologies [21]. RADAR-base provides both passive and active data collection via two applications – active and passive. The passive app collects real time monitoring of movement, location, audio and app usage [21]. The active app collects self-reported user questionnaires. Data from both apps are streamed in real-time to project servers. It is important to note that RADAR-base does not provide a feedback loop to the participant or any clinicians.

In total, 623 participants met the eligibility criteria and were recruited between November 2017 and June 2020 across three European countries: United Kingdom (*n* = 350; 56.2%), Spain (*n* = 155; 24.9%) and The Netherlands (*n* = 118; 18.4%). Participants in the UK and The Netherlands were recruited from community samples including individuals from existing studies on depression and using local clinical services. All participants recruited for this study had pre-existing major depressive disorder, and all recruitment sites utilised the same eligibility criteria for entry into the study. The Netherlands also recruited through advertisements in general practices and psychologist practices, newspaper advertisements and through Hersenonderzoek.nl (https://hersenonderzoek.nl). Spanish participants were recruited from a clinical sample of individuals seeking help for a mental health condition.

Each participant was asked to wear a wrist-worn activity tracker (FitBit Charge 2 or 3) and install the active and passive RADAR-base applications onto their smartphones (see [18, 21] for further details). The project was developed using co-design and in partnership with a Patient Advisory Group. Project apps were used to collect data passively from existing smartphone sensors, and to deliver questionnaires, cognitive tasks, and speech assessments. The wrist-worn activity tracker and project apps collected data on participants’ sleep, physical activity, stress, mood, self-esteem, sociability, speech patterns, and cognitive function.

The RADAR-MDD project is currently on-going and final data collection is expected in March 2021. Participants were excluded from the current study if they had withdrawn from the RADAR-MDD project at any time (*n* = 78; 12.5%), enrolled in RADAR-MDD after the 1st December 2019 (*n* = 200; 32.1%), had not completed a self-reported Patient Health Questionnaire (PHQ) in December 2019, or were missing basic demographics at baseline (*n* = 93; 14.9%). A total of 252 (40.5%) participants remained after exclusions and their data was used for analysis.

The RADAR-MDD project received ethical approval in the United Kingdom from the Camberwell St Giles Research Ethics Committee (REC reference: 17/LO/1154); and Spain from the CEIC Fundació Sant Joan de Déu (CI reference: PIC-128-17) and in The Netherlands from the Medische Ethische Toetsingscommissie VUmc (METc VUmc registratienummer: 2018.012 – NL63557.029.17). The research was undertaken in accordance with the Declaration of Helsinki, and all participants provided informed consent to participate.

### Measures and features

The RADAR-MDD project collects a range of validated measures from participants at different timepoints (see further [18] information) using the RADAR-base active app [25]. RADAR-base sends automatic survey invitations (email and in-app push notification).

#### Depressive symptoms

The Patient Health Questionnaire (PHQ-8 [26];) was delivered every 2 weeks via the project app. The PHQ-8 is an 8-item self-report questionnaire which measures the frequency of depressive symptoms over the preceding 2-week period. Each item is rated on a scale of 0–3, producing a range of total scores from 0 to 24. The PHQ-8 has good validity, reliability, sensitivity, and specificity in the general population [26]. In this study, a cut-off score of 10 or more is defined as a case of clinically relevant depressive symptoms (hereafter ‘depression’) [26].

#### Self-esteem

The Rosenberg Self-Esteem Scale (RSES [27];) was delivered every 2 weeks via the project app alongside the PHQ-8. The RSES is a 10-item self-report instrument for evaluating individual self-esteem [27–29]. Each item is rated on a scale of 1–3 (half the questions are reverse scored), producing a range of scores from 0 to 30. Scores between 15 and 25 are within normal range, with scores below 15 suggesting low self-esteem [27].

#### Sleep duration

Participants enrolled in the RADAR-MDD project were asked to wear a wrist-worn activity tracker (FitBit Charge 2 or 3) over the study duration as much as possible, including when sleeping. The device collected parameters on heart rate and sleep duration. In this study, total sleeping minutes, as computed by the FitBit Charge 2 or 3, was extracted for each participant for each day and a daily feature was calculated to represent the amount slept for each 24-h period. Total sleep duration was calculated between 8:00 pm (20:00) as the starting time point and 11:00 am (11:00) as the finishing timepoint (following a procedure reported previously [30]). Where no data was found due to the participant not wearing the device, no features were computed for that day.

### Data analysis

Socio-demographic characteristics were summarised using frequencies and unweighted percentages or medians with interquartile ranges (IQR) for the overall sample and for each country individually. Outcome variables (depressive symptoms, self-esteem and sleep duration were then summarised across three timepoints: pre-, during- and post-lockdown (defined as restriction easing in each country). A mean value was computed for depressive symptoms (PHQ-8 score), self-esteem (RSES score) and sleep duration (minutes) for each participant within each of these timepoints.

The following dates were used to define these timepoints [31]:

- United Kingdom: lockdown: 23/03/2020 and easing restrictions: 11/05/2020;
- Spain: lockdown: 14/03/2020 and easing restrictions: 02/05/2020;
- The Netherlands: lockdown: 17/03/2020 and easing restrictions: 11/05/2020.

Changes in the mean total score of each outcome variable over these timepoints were analysed using linear mixed models for repeated measures. Linear mixed models are a generalisation of linear regression which permit modelling of repeated measures data by incorporating a random effect of ‘participant’. First, we investigated the overall changes in each outcome variable using timepoint (pre-, during- and post-lockdown) as the exposure variable. We then added pre-pandemic depression caseness into each model as a second exposure variable, including an interaction term between timepoint and depression caseness, to investigate whether rate of change in the outcome variables over time varied according to depression caseness. Pre-pandemic depression caseness (denoted as: no depression, depression) was defined as a participant scoring 10 or more on the PHQ-8 during December 2019. This was used to define clinically relevant depressive symptoms shortly before the pandemic.

We used post-estimation commands to further explore the associations identified in mixed modelling. Models were fitted using Maximum Likelihood Estimation and an unstructured residual-error covariance matrix. Mixed models can produce valid estimates even when data is not missing completely at random, without the need for further missing data techniques like multiple imputation [32].

A participant could have completed a maximum of 18 PHQ-8/RSES self-report questionnaires during the analysis timepoints. RMT offers a unique ability to monitor and track participants, however due to the frequency of data collection, technical issues and daily life, missing data is inevitable, and further information relating to this is presented in Supplement A. Statistical significance was defined as a *p*-value of less than 0.05. Data processing was performed in Python version 3.5. All analyses were performed using STATA MP 16.1.

## Results

### Socio-demographic characteristics at baseline

The majority of the sample was female (*n* = 188; 74.6%), had clinically relevant depressive symptoms shortly before the pandemic (*n* = 121, 48.0%), was cohabiting or married (*n* = 138; 54.8%) and was on medication for management of depression (*n* = 166; 65.9%) at baseline (see Table 1).

**Table 1:** Cohort characteristics at baseline (*n* = 252) stratified by country

Variable	Overall (***n*** = 252)	United Kingdom (***n*** = 140; 55.6%)	Spain (***n*** = 70; 27.8%)	The Netherlands (***n*** = 42; 16.7%)
Sex				
Male	64 (25.4)	30 (21.3)	24 (34.3)	10 (23.8)
Female	188 (74.6)	110 (78.6)	46 (65.7)	32 (76.2)
Marital status				
Single	75 (29.8)	40 (28.6)	10 (14.3)	25 (59.5)
Married/cohabiting	138 (54.8)	82 (58.6)	43 (61.4)	13 (30.9)
Divorced/Separated/Widowed	39 (15.5)	18 (12.9)	17 (24.3)	4 (9.5)
Employment				
Employed	115 (45.6)	66 (47.1)	28 (40.0)	21 (50.0)
Retired	64 (25.4)	35 (25.0)	25 (35.7)	4 (9.5)
Student	23 (9.1)	12 (8.8)	1 (1.4)	10 (23.8)
Unemployed	26 (10.3)	14 (10.0)	9 (12.9)	3 (7.1)
Other	24 (9.5)	13 (9.3)	7 (10.0)	4 (9.5)
Age (in years)				
< 25	16 (6.4)	7 (5.0)	–	9 (21.4)
25–34	35 (13.9)	22 (15.7)	2 (2.9)	11 (26.2)
35–44	38 (15.1)	22 (15.7)	12 (17.1)	4 (9.5)
45–54	42 (16.7)	19 (13.6)	18 (25.7)	5 (11.9)
55–64	81 (32.2)	44 (31.4)	28 (40.0)	9 (21.4)
65>	40 (15.9)	26 (18.6)	10 (14.3)	4 (9.5)
Medication for Depression				
No	48 (19.1)	36 (25.7)	2 (2.9)	10 (23.8)
Yes	166 (65.9)	80 (57.1)	65 (92.9)	21 (50.0)
Not reported	38 (15.1)	24 (17.1)	3 (4.3)	11 (26.2)
Depression^a^ (December 2019)				
No Depression	131 (52.0)	87 (62.1)	28 (40.0)	16 (38.10)
Depression	121 (48.0)	53 (37.9)	42 (60.0)	26 (61.9)
Length of education (in years) (mean, SD)^b^	15.9 (6.5)	16.5 (5.5)	12.5 (4.9)	19.3 (8.9)
Length of time in study in days [median, IQR]^b^	253 (124 to 327)	285.5 (186.5 to 435)	257.5 (158 to 306)	109.5 (44 to 170)

^a^As measured by the Patient Health Questionnaire [26]. Depression defined as scoring 10 or more. ^b^Up to 1st December 2019

### Depressive symptom trajectories

Overall, mean depressive symptoms remained stable between pre- and during-lockdown (estimated mean score difference: -0.18; CI: − 0.61 to 0.24, *p* = 0.339) and between during- and post-lockdown (estimated mean score difference: -0.03; CI: − 0.42 to 0.36, *p* = 0.882) (Table 2).

**Table 2:** Estimated overall differences in each outcome variable between each timepoint. Results stratified by country are available from the corresponding author

	Estimated difference between pre- and during- lockdown (95% CI, *p*-value)	Estimated difference between during- and post- lockdown 95% CI, *p*-value)
Mean PHQ-8 score	-0.18 (− 0.61 to 0.24; *p* = 0.339)	-0.03 (− 0.42 to 0.36; *p* = 0.882)
Mean RSES score	-0.06 (− 0.22 to 0.10; *p* = 0.445)	0.07 (− 0.08 to 0.22; *p* = 0.381)
Mean sleep duration	-0.01 (− 5.55 to 5.56; *p* = 1.000)	-12.16 (− 18.39 to − 5.92; *p* < 0.001)

We then added an interaction term between depression caseness and timepoint to investigate whether these trajectories varied according to depression caseness. Perhaps unsurprisingly, those with pre-pandemic depression reported more depressive symptoms at all three timepoints (Table 3). However, there was also some evidence for an interaction between depression caseness and timepoint in predicting course of depressive symptoms between pre- and during-lockdown (*p* = 0.047; Table 3).

**Table 3:** Estimated difference in each outcome variable between no depression and depression (in December 2019) at each timepoint, and differences in rate of change over time. Results stratified by country are available from the corresponding author

	Pre-lockdown estimate	During-lockdown estimate	Post-lockdown estimate	Evidence for a difference in the rate of change between pre- and during-lockdown. (Interaction *p*-value)	Evidence for a difference in the rate of change between during- and post-lockdown. (Interaction *p*-value)
*n* = 252	(mean PHQ-8 score difference, 95% CI)	(mean PHQ-8 score difference, 95% CI)	(mean PHQ-8 score difference, 95% CI)		
No Depression	Reference group	–	–	–	–
Depression	9.33 (8.32 to 10.34)	8.47 (7.21 to 9.73)	7.83 (6.70 to 8.96)	0.047	0.112
*n* = 252	(mean RSES score difference, 95% CI)	(mean RSES score difference, 95% CI)	(mean RSES score difference, 95% CI)		
No Depression	Reference group	–	–	–	–
Depression	−1.09 (− 1.46 to −0.72)	− 1.43 (− 1.85 to − 1.05)	− 1.31 (− 1.69 to − 0.92)	0.045	0.461
*n* = 240	(mean sleep duration difference, 95% CI)	(mean sleep duration difference, 95% CI)	(mean sleep duration difference, 95% CI)		
No Depression	Reference group	–	–	–	–
Depression	−10.48 (−28.38 to 7.41)	−32.98 (−53.32 to − 12.64)	−28.26 (−50.67 to −5.85)	< 0.001	0.458

**Fig. 1:**
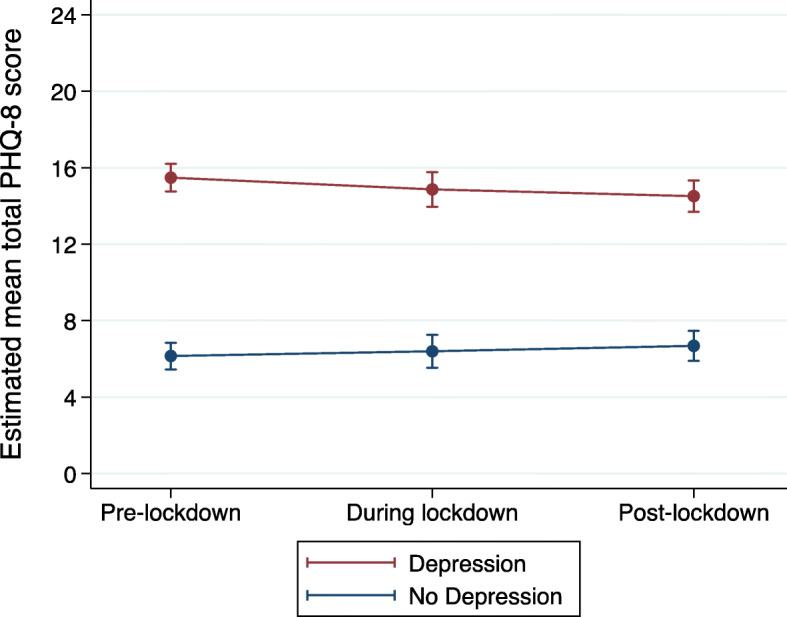
Mean PHQ-8 score trajectories by depression caseness, as estimated from the repeated measures mixed model

We further investigated this using post-estimation commands and found very weak evidence that the depressed group showed a decrease in depressive symptoms between pre- and during-lockdown (estimated mean score: -0.61; CI: − 1.23 to 0.01; *p* = 0.051), whereas the non-depressed group remained stable (estimated mean score: 0.24; CI: − 0.33 to 0.83, *p* = 0.409) (Fig. 1).

### Self-esteem trajectories

Overall, mean self-esteem score remained stable between pre- and during-lockdown (estimated mean score difference: -0.06; CI: − 0.22 to 0.10, *p* = 0.445) and between during- and post-lockdown (estimated mean score difference: 0.07; CI: − 0.08 to 0.22, *p* = 0.381) (Table 2).

We then added an interaction term between depression caseness and timepoint to investigate whether these trajectories varied according to depression caseness. Those with pre-pandemic depression reported lower self-esteem scores throughout the pandemic than those without depression (Table 3). There was also some evidence for an interaction between depression caseness and timepoint in predicting course of self-esteem between pre- and during-lockdown (*p* = 0.045; Table 3).

**Fig. 2:**
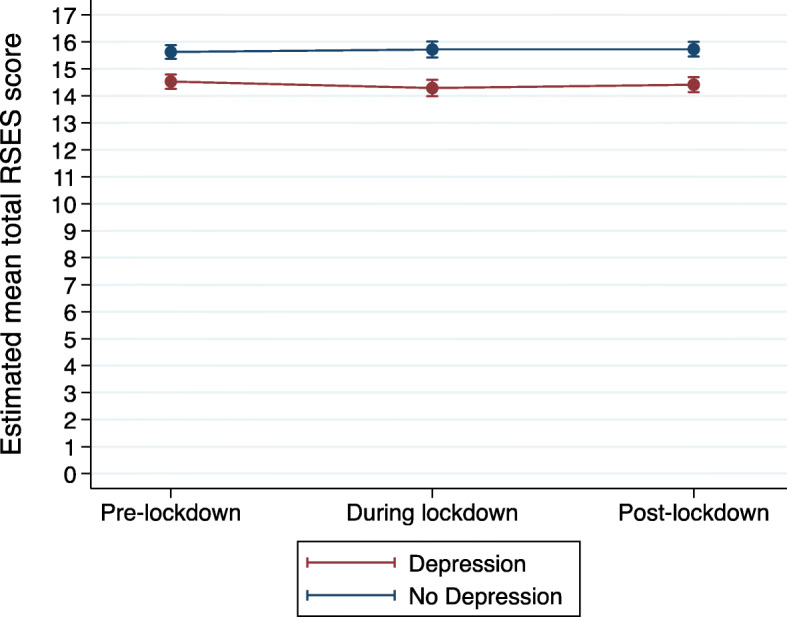
Mean RSES score trajectories by depression caseness, as estimated from the repeated measures mixed model

We further investigated this using post-estimation commands and found evidence that the depressed group showed reducing self-esteem scores between pre- and during-lockdown (estimated mean score: -0.24; CI: − 0.47 to 0.01; *p* = 0.048), whereas the non-depressed group remained stable (estimated mean score: 0.09; CI: − 0.13 to 0.32, *p* = 0.409) (Fig. 2).

### Sleep duration trajectories

Overall, mean sleep duration remained stable between pre- and during-lockdown (estimated mean duration difference: -0.01; CI: 5.55 to 5.56, *p* = 1.000). However, between during- and post-lockdown there was evidence of a significant decrease in mean sleep duration (estimated mean duration difference: -12.16; CI: − 18.39 to − 5.92, *p* < 0.001) (Table 2).

We then added an interaction term between depression caseness and timepoint to investigate whether these trajectories varied according to depression caseness. Those with pre-pandemic depression reported shorter sleep durations during- and post-lockdown relative to those without (Table 3). There was also evidence for an interaction between depression caseness and timepoint in predicting course of mean sleep duration between pre- and during-lockdown (*p* < 0.001; Table 3).

**Fig. 3:**
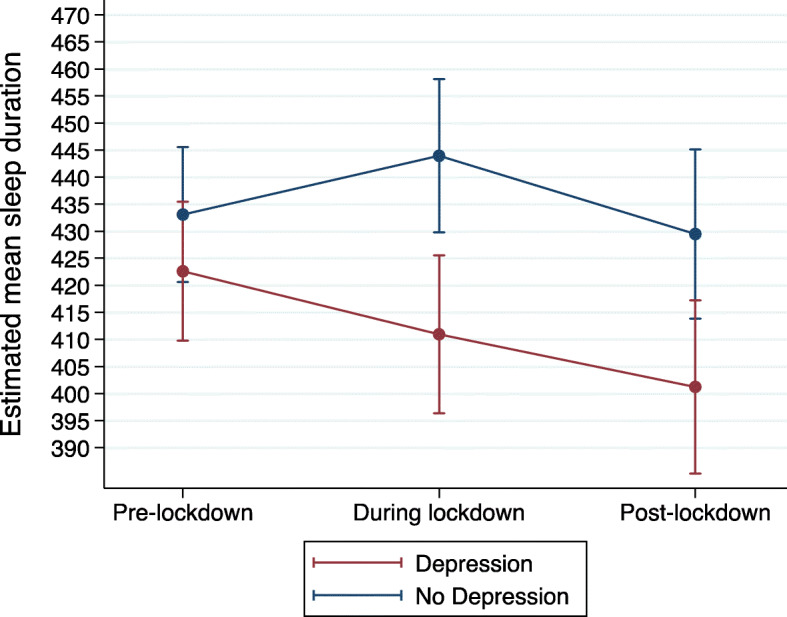
Mean sleep duration (in minutes) trajectories by depression caseness, as estimated from the repeated measures mixed model

We further investigated this using post-estimation commands and found strong evidence that the depressed group showed significant decreases in mean sleep duration between pre- and during-lockdown (estimated mean duration difference: -11.64; CI: − 19.33 to − 3.95; *p* = 0.003), whereas the non-depressed group significantly increased mean sleep duration (estimated mean sleep duration difference: 10.85; CI: 3.43 to 18.27; *p* = 0.004) (Fig. 3). However, the interaction between depression caseness and timepoint between during- and post-lockdown was not statistically significant, suggesting that both depression and no depression groups showed a similar rate of decline in sleep duration between these timepoints.

## Discussion

In this study, we investigated the depressive symptom trajectories for a cohort of adults with a recent history of MDD. For the sample as a whole, we found no evidence that depressive symptoms or self-esteem changed over the course of lockdown. However, we found evidence that mean sleep duration decreased between during- and post- lockdown. We also found that, relative to those who did not show evidence of clinically relevant depressive symptom severity shortly before the pandemic, those with pre-pandemic depression showed a significant decrease in sleep duration (in minutes) between pre- and during- lockdown. However, while there were also reductions in symptom and self-esteem scores, this reduction was not clinically meaningful.

The Covid-19 pandemic represents a unique health, social and economic challenge, with the impact on global mental health expected to be high [33], the use of RMT to explore a pre-existing MDD cohort has provided unique insights into behaviours over the duration of the pandemic. The rapid spread and persistence of Covid-19 has increased health anxieties, and has resulted in an increase in mental health disorders globally [34]. In our study, we focused on a less studied area, those with pre-existing MDD, which has been shown to be negatively impacted as a result of the Covid-19 pandemic, with the severity varying based on occupation, gender, geographical location and physical/mental health comorbidities [33, 35, 36]. There are major differences in the prevalence of depression globally, with one US cohort identifying a three-fold increase in depression symptoms during the pandemic than before [35]. This contrasts a Dutch study, which found that while those with depression scored highly on symptom scales, they did not report greater increases in symptoms during the pandemic [37].

Our overall findings are similar to those reported in The Netherlands, while overall depression score changed compared with a period just before the pandemic, the change was not clinically meaningful and suggests participants depression remained stable. It is important to acknowledge that lockdown restrictions could have moderated depression symptom change, with varying levels of restrictions imposed across the UK, The Netherlands and Spain. In addition, our findings could be explained as regression to the mean, or improvements in depression over time due to the cyclic nature of the disorder.

Sufficient sleep is essential for cognitive and psychological functioning, and prior research has shown that poor quality sleep is associated with depression and longer term health outcomes [38–40]. Using data collected via a wearable device and RMT, we found that trajectories of sleep duration decreased significantly pre-, during- and post-lockdown in the depressed group with evidence of an interaction between pre- and during- lockdown. This reduction in sleep, and persistence to post-lockdown could indicate an adverse impact of the pandemic and requires further investigation to assess its impact on clinical outcomes. An alternative theory could be that reduced activity levels could result in less sleep being required, and not, therefore, detrimental but merely an adjustment in how much sleep is needed. It must be acknowledged that prior research has indicated that FitBit devices underestimate sleep onset, which could have impacted our results [41].

Self-esteem is an important indicator of self-worth. When a participant has healthy self-esteem, they tend to feel positive about themselves, and about life in general. Conversely, when self-esteem is low, participants tend to see themselves and life in a more negative and critical light [27, 29, 42]. In this study, we found that there were differences in self-esteem across timepoints, with small but not clinically meaningful changes between pre-, and during- lockdown. This could suggest an overall small change in the outlook of participants with pre-pandemic depression.

Our study demonstrates the utility of RMT and wearable technology to evaluate the impact of Covid − 19. The potential to gather high-frequency information via wearable technology and smartphones using platforms such RADAR-MDD project has advantages over momentary data capture currently employed in a clinical setting, which often presents in the form of infrequent questionnaires. The capacity of RMT to offer passive monitoring, without the need for active participant engagement, allows longitudinal assessment of more objectively collected outcomes as well as the capacity to offer multi-parametric monitoring. The results of this work enhance our understanding of the impact of lockdown due to Covid − 19 for those with MDD as monitored through RMT.

The Covid-19 pandemic presents a unique opportunity for deploying RMT to track disease progression or relapse in symptoms, despite the global lockdown and frequent local restrictions. This works demonstrates the application of large-scale multi-parametric monitoring, gathered at an incomparable frequency to once-off clinic visits, providing real-time data on the effects of Covid-19 for those with MDD and their environment. The unique strength of this work is the ability to directly compare results gathered during- and post-lockdown with previously collated shortly before the start of the pandemic. Many studies rely on participant recall of pre-pandemic health and well-being statuses for comparison purposes. The nature of this study avoids potential recall bias, in addition to the fact that symptom changes are tracked at a high frequency and responses are submitted in real-time via a phone application. The original study aims were not related to Covid − 19, and participants included in these analyses joined before December 2019, which allows for the elimination of lockdown bias; participants were not motivated to take part simply on the basis of being personally affected by the pandemic.

Notwithstanding these strengths, our study does have some limitations. First, technical issues with mobile devices and RMT apps could have impacted data collection and have contributed to missing data. In addition, participant adherence to the study protocol (ie. questionnaire completions) decreases over time which further contributes to missing data. While it is difficult to overcome these issues, it is more reflective of real-world data collection and future work will explore if missing data, in itself, is an informative indicator of the participant’s current state. In addition, the pandemic has placed unique stressors on individuals which may have further contributed to data completes. For example, some participants may have been more adherent to the RADAR-MDD project due to fewer life distractions, whereas in some life could have become very difficult resulting in less adherence. Future research should seek to explore this further. Second, RADAR-MDD project used a range of recruitment strategies across the different sites including self-enrolment registers and clinical services, therefore the sample is not necessarily representative of the wider population with MDD. Third, when assessing sleep duration, this study only considered those who slept during the night and not those who had daytime sleep duration. Further work should be undertaken to explore differences between daytime sleep and night-time sleep duration.

Global healthcare systems face unprecedented challenges in meeting the unmet mental health need. The effects of this pandemic will undoubtedly have far-reaching consequences, with potential long and short-term psychological impacts. However, the results of this study suggest that those with MDD do not experience a significant worsening in symptoms during the first months of the Covid − 19 pandemic. Future work should attempt to examine the association between individual socio-demographic characteristics and changes in the examined variables, notable prior to the pandemic and within an individual, to further unpick the impact on those with MDD.

## Supplementary Information

**Additional file 1 **: **Supplement A: Missing Data**.

## Data Availability

The datasets generated and/or analysed during the current study are not publicly available due to on-going data collection but are available from the corresponding author on reasonable request.

## Abbreviations

IQR: Interquartile Range
MDD: Major Depressive Disorder
PHQ8: Patient Health Questionnaire
RADAR-MDD: Remote Assessment of Disease and Relapse in individuals with Major Depressive Disorder
RMT: Remote Measurement Technology
RSES: Rosenberg Self Esteem Scale
SARS-Cov-2: Severe acute respiratory syndrome coronavirus 2
WHO: World Health Organisation

## References

[1] 2019 Novel Coronavirus (2019‐nCoV): Strategic preparedness and response plan [Internet]. Geneva, Switzerland; 2020. Available from: https://www.who.int/publications/i/item/strategic-preparedness-and-response-plan-for-the-new-coronavirus.

[2] Zhu N, Zhang D, Wang W, Li X, Yang B, Song J (2020). A novel coronavirus from patients with pneumonia in China, 2019. N Engl J Med [internet].

[3] Petersen E, Koopmans M, Go U, Hamer DH, Petrosillo N, Castelli F (2020). Comparing SARS-CoV-2 with SARS-CoV and influenza pandemics. Lancet Infect Dis [Internet].

[4] Ghebreyesus TA. WHO director-General’s opening remarks at the media briefing on COVID-19 - 11 may 2020 [internet]. 2020. Available from: https://www.who.int/dg/speeches/detail/who-director-general-s-opening-remarks-at-the-media-briefing-on-covid-19---11-may-2020.

[5] Hotopf M, Bullmore E, O’Connor RC, Holmes EA (2020). The scope of mental health research during the COVID-19 pandemic and its aftermath. Br J Psychiatry.

[6] Pierce M, Hope H, Ford T, Hatch S, Hotopf M, John A (2020). Mental health before and during the COVID-19 pandemic: a longitudinal probability sample survey of the UK population. Lancet Psychiatry.

[7] Brooks SK, Webster RK, Smith LE, Woodland L, Wessely S, Greenberg N (2020). The psychological impact of quarantine and how to reduce it: rapid review of the evidence. Lancet..

[8] Brunier A. COVID-19 disrupting mental health services in most countries, WHO survey [Internet]. World Health Organization. 2020 [cited 2021 Aug 27]. Available from: https://www.who.int/news/item/05-10-2020-covid-19-disrupting-mental-health-services-in-most-countries-who-survey.

[9] Holmes EA, O’Connor RC, Perry VH, Tracey I, Wessely S, Arseneault L (2020). Multidisciplinary research priorities for the COVID-19 pandemic: a call for action for mental health science. Lancet Psychiatry.

[10] Xiang Y-T, Yang Y, Li W, Zhang L, Zhang Q, Cheung T (2020). Timely mental health care for the 2019 novel coronavirus outbreak is urgently needed. Lancet Psychiatry.

[11] Vindegaard N, Benros ME (2020). COVID-19 pandemic and mental health consequences: systematic review of the current evidence. Brain Behav Immun.

[12] Kwong ASF, Pearson RM, Adams MJ, Northstone K, Tilling K, Smith D (2021). Mental health before and during the COVID-19 pandemic in two longitudinal UK population cohorts. Br J Psychiatry.

[13] Falkingham J, Evandrou M, Qin M, Vlachantoni A. “Sleepless in Lockdown”: Unpacking Differences in Sleep Loss During the Coronavirus Pandemic in the UK. SSRN Electron J. 2020; 10.1136/bmjopen-2021-053094 PMC8724580 34980617

[14] Li S, Chan J, Lam J, Yu M, Wing Y (2013). Can nocturnal sleep disturbances predict non-remission and relapse in patients with major depressive disorder? V a 5-year naturalistic longitudinal study. Sleep Med.

[15] Shallcross AJ, Gross JJ, Visvanathan PD, Kumar N, Palfrey A, Ford BQ (2015). Relapse prevention in major depressive disorder: mindfulness-based cognitive therapy versus an active control condition. J Consult Clin Psychol.

[16] van Rijsbergen GD, Bockting CLH, Burger H, Spinhoven P, Koeter MWJ, Ruhé HG (2013). Mood reactivity rather than cognitive reactivity is predictive of depressive relapse: a randomized study with 5.5-year follow-up. J Consult Clin Psychol.

[17] The Mobile Economy Europe [Internet]. 2021 [cited 2021 Feb 22]. Available from: https://www.gsma.com/mobileeconomy/europe/

[18] Matcham F, Barattieri di San Pietro C, Bulgari V, de Girolamo G, Dobson R, Eriksson H (2019). Remote assessment of disease and relapse in major depressive disorder (RADAR-MDD): a multi-centre prospective cohort study protocol. BMC Psychiatry.

[19] Velupillai S, Hadlaczky G, Baca-Garcia E, Gorrell GM, Werbeloff N, Nguyen D (2019). Risk assessment tools and data-driven approaches for predicting and preventing suicidal behavior. Front Psychiatry.

[20] Wickersham A, Petrides PM, Williamson V, Leightley D (2019). Efficacy of mobile application interventions for the treatment of post-traumatic stress disorder: a systematic review. Digit Heal.

[21] Ranjan Y, Rashid Z, Stewart C, Conde P, Begale M, Verbeeck D (2019). RADAR-base: open source Mobile health platform for collecting, monitoring, and analyzing data using sensors, wearables, and Mobile devices. JMIR mHealth uHealth..

[22] Campion J, Javed A, Sartorius N, Marmot M (2020). Addressing the public mental health challenge of COVID-19. Lancet Psychiatry.

[23] Xiong J, Lipsitz O, Nasri F, Lui LMW, Gill H, Phan L (2020). Impact of COVID-19 pandemic on mental health in the general population: a systematic review. J Affect Disord.

[24] Liu Q, He H, Yang J, Feng X, Zhao F, Lyu J (2020). Changes in the global burden of depression from 1990 to 2017: findings from the global burden of disease study. J Psychiatr Res.

[25] Harris PA, Taylor R, Thielke R, Payne J, Gonzalez N, Conde JG (2009). Research electronic data capture (REDCap)—a metadata-driven methodology and workflow process for providing translational research informatics support. J Biomed Inform.

[26] Kroenke K, Strine TW, Spitzer RL, Williams JBW, Berry JT, Mokdad AH. The PHQ-8 as a measure of current depression in the general population. J Affect Disord 2009;114(1–3):163–73. 10.1016/j.jad.2008.06.026 18752852

[27] Greenberger E, Chen C, Dmitrieva J, Farruggia SP (2003). Item-wording and the dimensionality of the Rosenberg self-esteem scale: do they matter?. Pers Individ Dif.

[28] Sinclair SJ, Blais MA, Gansler DA, Sandberg E, Bistis K, LoCicero A (2010). Psychometric properties of the Rosenberg self-esteem scale: overall and across demographic groups living within the United States. Eval Health Prof.

[29] Fleming JS, Courtney BE (1984). The dimensionality of self-esteem: II. Hierarchical facet model for revised measurement scales. J Pers Soc Psychol.

[30] Sun S, Folarin AA, Ranjan Y, Rashid Z, Conde P, Stewart C (2020). Using smartphones and wearable devices to monitor behavioral changes during COVID-19. J Med Internet Res.

[31] Kantis C, Kiernan S, Bardi Socrates J. Timeline of the Coronavirus [Internet]. Think Global Health. 2020 [cited 2020 Jun 21]. Available from: https://www.thinkglobalhealth.org/article/updated-timeline-coronavirus

[32] Detry MA, Ma Y (2016). Analyzing Repeated Measurements Using Mixed Models. JAMA [Internet].

[33] Bueno-Notivol J, Gracia-García P, Olaya B, Lasheras I, López-Antón R, Santabárbara J (2020). Prevalence of depression during the COVID-19 outbreak: a meta-analysis of community-based studies. Int J Clin Heal Psychol.

[34] Salari N, Hosseinian-Far A, Jalali R, Vaisi-Raygani A, Rasoulpoor S, Mohammadi M (2020). Prevalence of stress, anxiety, depression among the general population during the COVID-19 pandemic: a systematic review and meta-analysis. Glob Health.

[35] Ettman CK, Abdalla SM, Cohen GH, Sampson L, Vivier PM, Galea S (2020). Prevalence of depression symptoms in US adults before and during the COVID-19 pandemic. JAMA Netw Open.

[36] Wickersham A, Carr E, Hunt R, Davis JP, Hotopf M, Fear NT (2021). Changes in Physical Activity among United Kingdom University Students Following the Implementation of Coronavirus Lockdown Measures. Int J Environ Res Public Health [Internet].

[37] Pan K-Y, Kok AAL, Eikelenboom M, Horsfall M, Jörg F, Luteijn RA (2021). The mental health impact of the COVID-19 pandemic on people with and without depressive, anxiety, or obsessive-compulsive disorders: a longitudinal study of three Dutch case-control cohorts. Lancet Psychiatry.

[38] Majumdar P, Biswas A, Sahu S (2020). COVID-19 pandemic and lockdown: cause of sleep disruption, depression, somatic pain, and increased screen exposure of office workers and students of India. Chronobiol Int.

[39] Marelli S, Castelnuovo A, Somma A, Castronovo V, Mombelli S, Bottoni D (2021). Impact of COVID-19 lockdown on sleep quality in university students and administration staff. J Neurol.

[40] Cellini N, Canale N, Mioni G, Costa S (2020). Changes in sleep pattern, sense of time and digital media use during COVID-19 lockdown in Italy. J Sleep Res.

[41] Liang Z, Chapa-Martell MA (2019). Accuracy of Fitbit wristbands in measuring sleep stage transitions and the effect of user-specific factors. JMIR mHealth uHealth.

[42] Orth U, Robins RW (2013). Understanding the link between low self-esteem and depression. Curr Dir Psychol Sci.

