# Supplementary material for: Investigating the impact of COVID-19 lockdown on adults with a recent history of recurrent major depressive disorder: a multi-Centre study using remote measurement technology
Source: BMC Psychiatry. 2021 Sep 6;21:435. doi: 10.1186/s12888-021-03434-5 (PMC8419819; doi:10.1186/s12888-021-03434-5)
Supplement: Supplementary file 1 — Additional file 1 : Supplement A: Missing Data. [file 12888_2021_3434_MOESM1_ESM.docx]

**Supplement A: Missing Data**

| **Period/Data Point** | Patient Health Questionnaire (8-item) | Rosenberg Self-Esteem Scale | Sleep duration (as collected via FitBit Charge 2 or 3) |
| --- | --- | --- | --- |
|  | n missing (%) | n missing (%) | n missing (%) |
| **December 2019 (Reference)** | 0 (%) | - | - |
| **Pre-lockdown** | 9 (5.6) | 9 (5.6) | 45 (17.6) |
| **During-lockdown** | 33 (13.1) | 34 (13.5) | 58 (23.1) |
| **Post-lockdown** | 29 (11.5) | 30 (11.9) | 76 (30.2) |

S-Table 1: Missing data for the cohort (total sample=252). Reported as the number of participants who have no data for a period.
